# Concerns with attempts by neuroeconomics to answer the philosophical question “Is it rational to donate money for charity?”

**DOI:** 10.3389/fpsyg.2013.00585

**Published:** 2013-08-30

**Authors:** Sumitava Mukherjee

**Affiliations:** Indian Institute of Technology GandhinagarAhmedabad, Gujarat, India

**Keywords:** donations, altruism, neuroeconomics, rational, brain mapping

Altruism involves acting toward the welfare of others by incurring costs to the self (Batson and Shaw, 1991). The theoretical question is: Why would people help others who are unrelated genetically? This was considered a “large anomaly” from rationality by neo-classical economics (Fehr and Fischbacher, 2003) as humans are assumed to be maximizers of decision utility—a purely selfish “homo-economicus” who should not spend on unrelated others. However, money given to the poor could create a private good for the recipient but at the same time, it creates a non-rival non-excludable benefit (or utility) for all those citizens who value a condition where poor people are also well fed and can live a decent life (Mayr et al., 2009). Thus, there could be a “warm glow” (Andreoni, 1995) by giving to charity (“impure altruism”).

Harbaugh et al. (2007) designed a novel experiment which strikingly showed that both mandatory and voluntary transfers to charity show neural activity in the brain areas associated with reward processing with larger activations following voluntary transfers. These results support both pure and impure forms of altruism. The brain areas implicated were medial orbitofrontal cortex (OFC) and the dorso-lateral prefrontal cortex (DLPFC) along with the ventral striatum, nucleus accumbens (NAcc) and the insulae which form parts of the reward circuitry that codes most real life rewards (like money, drugs and chocolates). The authors later argued that these results show donating to charity is inherently rewarding and rational (Mayr et al., 2009).

The reasoning adopted was: If brain area B is involved in coding rewards which have inherent utility and then we observe area B is involved in donations, then there is utility in donating and hence donating is rational (see Mayr et al., 2009). Is this conclusion warranted from the current data?

This line of reasoning uses reverse inference—a widespread but questionable technique (Poldrack, 2006) in neuroeconomics which infers mental states from brain states. The conclusions could be flawed under the assumptions of functionalism as multiple brain states can map to multiple mental states in a non-exclusive manner (Levin and Aharon, 2011).

In fact, multiple studies highlight the same brain areas implicated by Harbaugh and colleagues for other cognitive processes apart from donations. For example, medial frontal and posterior cingulate are also involved in moral decision making (Greene et al., 2001) while ventral OFC represent outcome values of primary reinforcers and abstract rewards (Peters and Büchel, 2010). Experienced utility and predictive error involved in both monetary and social reward tasks activate the vMPFC and straitum (Lin et al., 2012). It is now commonly accepted that generally the vMPFC is involved in valuation and a modulation of these signals is performed in the DLPFC which is a seat of self-control and emotion regulation (Hare et al., 2008). Thus, even though I do not attempt to provide a comprehensive review, it is obvious that the problem of inferring whether donating is a rational act by observing activations of the reward circuitry is not easy. Note that in an experiment even if a brain area (meso-limbic reward circuit) is active for both monetary rewards and donations (Moll et al., 2006), we cannot conclude that donating *is* rewarding or rational by itself or that there is a utility in donating.

Part of the theoretical confusion is due to multiple usages of “utility.” If one uses the concept of decision utility based on economical grounds, then charity might seem irrational (Fehr and Fischbacher, 2003). However, if one uses the hedonic form where utility stems from maximizing pleasures for oneself (“experienced utility”; Kahneman et al., 1997) then both pure altruism and warm-glow could have utility, since both maximize pleasures of seeing somebody gain (pure altruism) or of feeling good oneself (warm-glow).

The other important demonstration by Harbaugh et al. (2007) was that neural responses (in the reward circuit including the NAcc) to pure charity gains after subtracting responses to pure personal gains predict the rate of accepting altruistic transfers to some extent (27%). Whether responding to gains to oneself and gains to an organization neutrally code the same process is not clear. Hence, what a subtraction between these two conditions means is likewise not clear. As activations in the NAcc can increase not only for anticipated gains, but also for anticipated losses (Carter et al., 2009), it is possible that activations for charity gains can also be construed as personal losses. Also, note that the prediction is constructed from six different brain regions (averaged together) that in a way supposes that all these areas encode value, but what kind of utility is encoded and what computations are performed? It is of course possible that monetary gains to oneself and a charity are both valuable because both increase experienced utility. Crucially, future studies need to add more clarity about the motivational relevance of monetary transfers, definitions of utility being referred to and associated pre-suppositions.

Further, in general, what is meant by rational is often not explicit. If “rational” is to desire more of “good things,” it can be seen as experienced utility and if we take the normative route, it is confined to decision utility. But, is it epistemic or instrumental rationality? (Kelly, 2003). Even though rationality is taken as a starting point in economic analysis it is often not clear why that should be so (Foley, 2004). In fact, Foley argues that this supposes an imposition of modern capitalist social understanding which is simply one way society functions. Finally, if moral requirements instigate donating to social causes (Kant's categorical imperative; Johnson, 2012) or altruism is rational in itself (Nagel, 1978), then doesn't donating become rational by definition? These questions require more work before making convincing philosophical or theoretical deductions related to the rational basis of altruism.

I do not intend to suggest that the reasoning is wrong; but, conclusions from neuroscientific works are often based on many undisclosed assumptions and incomplete ontology, which requires more criticality to settle philosophical questions.
